# Respiratory Mechanics and Plasma Levels of Tumor Necrosis Factor Alpha and Interleukin 6 Are Affected by Gas Humidification during Mechanical Ventilation in Dogs

**DOI:** 10.1371/journal.pone.0101952

**Published:** 2014-07-18

**Authors:** Claudia Hernández-Jiménez, Rogelio García-Torrentera, J. Raúl Olmos-Zúñiga, Rogelio Jasso-Victoria, Miguel O. Gaxiola-Gaxiola, Matilde Baltazares-Lipp, Luis H. Gutiérrez-González

**Affiliations:** 1 Department of Experimental Surgery, Instituto Nacional de Enfermedades Respiratorias “Ismael Cosío Villegas”, Mexico City, Mexico; 2 Respiratory Therapy Service, Instituto Nacional de Enfermedades Respiratorias “Ismael Cosío Villegas”, Mexico City, Mexico; 3 Laboratory of Morphology, Instituto Nacional de Enfermedades Respiratorias “Ismael Cosío Villegas”, Mexico City, Mexico; 4 Department of Virology and Mycology, Instituto Nacional de Enfermedades Respiratorias “Ismael Cosío Villegas”, Mexico City, Mexico; The Ohio State University, United States of America

## Abstract

The use of dry gases during mechanical ventilation has been associated with the risk of serious airway complications. The goal of the present study was to quantify the plasma levels of TNF-alpha and IL-6 and to determine the radiological, hemodynamic, gasometric, and microscopic changes in lung mechanics in dogs subjected to short-term mechanical ventilation with and without humidification of the inhaled gas. The experiment was conducted for 24 hours in 10 dogs divided into two groups: Group I (n = 5), mechanical ventilation with dry oxygen dispensation, and Group II (n = 5), mechanical ventilation with oxygen dispensation using a moisture chamber. Variance analysis was used. No changes in physiological, hemodynamic, or gasometric, and radiographic constants were observed. Plasma TNF-alpha levels increased in group I, reaching a maximum 24 hours after mechanical ventilation was initiated (ANOVA p = 0.77). This increase was correlated to changes in mechanical ventilation. Plasma IL-6 levels decreased at 12 hours and increased again towards the end of the study (ANOVA p>0.05). Both groups exhibited a decrease in lung compliance and functional residual capacity values, but this was more pronounced in group I. Pplat increased in group I (ANOVA p = 0.02). Inhalation of dry gas caused histological lesions in the entire respiratory tract, including pulmonary parenchyma, to a greater extent than humidified gas. Humidification of inspired gases can attenuate damage associated with mechanical ventilation.

## Introduction

Mechanical ventilation (MV) is used in patients with respiratory failure and during general anesthesia. Under natural ventilation conditions, inhaled air is conditioned during its passage through the airways. When it reaches the alveoli, the air is fully saturated with water and is at body temperature (37°C, 100% relative humidity). The humidity/temperature requirements are not met when an artificial airway such as an endotracheal tube or tracheostomy cannula is used. Thus, an external humidifying device is usually added to the ventilator circuit. However, humidification is not general in developing countries. Patients subjected to MV lose the natural warming and humidifying functions for inhaled gases in the upper airway, which increases the risk of serious airway complications, including alterations in mucociliary transport, thickening of secretions, clogging of the airway with mucus, and ciliary dyskinesia. These complications can lead to hypothermia, hypoxemia, atelectasis, and inflammation [1], [2], [3]. Additionally, inflammation can promote the release of inflammatory mediators, resulting in biotrauma. Inflammatory mediators act systemically and can cause damage to other organs [4], [5]. Therefore, to maintain airway integrity, preserve mucociliary function, and improve gas exchange, inhaled gases should be humidified and warmed during MV with tracheal intubation [6], [7], [8].

It has been shown that MV is associated with the release of specific pro-inflammatory mediators, such as tumor necrosis factor alpha (TNF-α) and interleukin 6 (IL-6) [9], [10]. The goal of the present study was to quantify the plasma levels of TNF-α and IL-6 and to determine the radiological, hemodynamic, gasometric, and microscopic changes, as well as the modifications of lung mechanics in dogs subjected to short-term MV with and without humidification of the inhaled gas. It is known that the cellular lesion induced by dry gases may cause inflammation; it is also important, then, to determine how short-term dry air-administration has an effect not only at the cellular level, but on respiratory physiology in dogs.

## Materials and Methods

### Experimental animals

This experimental study was made in the Department of Experimental Surgery, National Institute of Respiratory Diseases “Ismael Cosío Villegas” (INER). Ten healthy crossbred adult dogs of both sexes weighing between 18 and 20 kg were used. Prior to the experiment the animals were confined in individual cages (1.0 m wide ×3.5 m long ×2.7 m high) [11] with the same environmental conditions. The animals had water and food *ad libitum*. This protocol was reviewed and approved by the Bioethics Committee of the INER (protocol number: B29-10). All animals were treated in strict accordance with the Technical Specifications for the Care and Use of Laboratory Animals of the Mexican Official Standard NOM-062-ZOO-1999 [11] and the Guide for the Care and Use of Laboratory Animals of the United States of America [12]. The sample size was reduced in agreement with the principles of experimental techniques proposed by Balls [13] and Kilkenny et al. [14]. The dogs were euthanized according to the Mexican Official Standard NOM-062-ZOO-1999 [11].

### Study groups

Ten animals were divided into two study groups and subjected to assisted MV for 24 hours.

GROUP I (n = 5): MV with dry oxygen dispensation.

GROUP II (n = 5): MV with oxygen dispensation using a humidification chamber.

### Experimental model

Anesthesia was induced with 6 mg/kg IV of propofol (Diprivan, Milan, Italy). The dogs were placed on a surgery table in dorsoventral position and were intubated with an endotracheal tube (Baxter, California, USA) connected to an Engstrom Carestation ventilator (General Electric, Madison, WI, USA). The device was set to ventilator mode, in which it delivers pressure-controlled ventilation with volume guarantee. To maintain the animals under anesthesia for 24 hours, an isofluorane Isotec 3 Ohmeda vaporizer (PISA Laboratories, Jalisco, Mexico) was used. Vital signs were monitored by electrocardiography (Datascope Passport, Paramus, NJ, USA). For the animals in group II, a MR850 humidity chamber (Fisher and Paykel, Auckland, New Zealand) with automatic compensation set to 37°C and with heater wire breathing circuit RT204 (Fisher and Paykel, Auckland, New Zealand) was used. Inspired gas, ambient air, and output ventilator temperatures were kept constant at 22°C [15]. A FiO_2_ between 0.25 and 0.30 was used. Positive end-expiratory pressure (PEEP) was maintained at 4 cm H_2_O. A tidal volume of 10 ml/kg was used.

### Assessment

The study was conducted for 24 hours. The following hemodynamic and gasometric parameters were assessed: cardiac frequency, cardiac output (determined using the thermodilution method [Hemodynamic Profile Computer Spectramed model SP-1445, Oxnard, USA]), mean arterial pressure, mean pulmonary artery pressure, pulmonary artery occlusion pressure, pulmonary vascular resistance, systemic vascular resistance, and shunt (Qs/Qt). Arterial (PaO_2_) and venous (PvO_2_) oxygen pressure, arterial (PaCO_2_) and venous (PvCO_2_) carbon dioxide pressure, and pH were measured with an ABL 800 Flex Analyzer (Radiometer, Brønshøj, Denmark).

### Radiography

Radiographic images of the animals in ventrodorsal position were taken prior to beginning mechanical ventilator assistance and at the end of the study.

### TNF-α and IL-6 quantification

Plasma cytokine levels were analyzed in triplicate using an enzyme-linked immunosorbent assay (ELISA), at t = 0, 12, and 24 hours. Canine TNF-α and IL-6 were detected with the Uscn Life Science Inc. E90133Ca and EIAab Science Co. detection kits, respectively. The sensitivity for TNF-α was 5.3 pg/mL, and the sensitivity for IL-6 was 15.6 pg/mL.

### Ventilation mechanics

Compliance (Cstat), airway resistance (Raw), plateau pressure (Pplat), peak inspiratory pressure (PIP), and mean airway pressure (Paw) were measured every two hours. Functional residual capacity (FRC) was determined at the beginning of the study and at 12 and 24 hours. The FRC was calculated based on the nitrogen elimination method by changing the flow of the oxygen/air concentration administered to the animals through the ventilator. The FRC value was calculated using a D-lite sensor and an airway module with energy output capacities (E-COVX, E-CAiOVX, M-COVX, M-CAiOVX). Briefly, an FRC procedure requires two measurements. At the beginning, the system records the N_2_ baseline concentration and changes the O_2_ adjustment to the O_2_ FRC adjustment. After a few breaths are taken, curve tracing begins. One measurement requires approximately 20 breaths. After the first measurement, the N_2_ and O_2_ concentrations are recorded, and the O_2_ is adjusted to the original value. The second FRC value appears when the second measurement is finished.

### Microscopic assessment

Tracheal, bronchial, main carina and pulmonary tissue samples were obtained and stained with hematoxylin and eosin (H&E). The sections were analyzed by light microscopy to determine the presence of the following: edema formation; hemorrhage; neutrophil, lymphocyte, and macrophage infiltration; and necrosis. The results were classified into four grades: grade 0 =  normal histology, grade 1 =  mild lesion, grade 2 =  moderate lesion, and grade 3 =  severe lesion [16].

### Statistical analysis

The data are expressed as the mean ± standard deviation. Variance analysis was used to determine the significance level. The Pearson correlation was used to analyze the association between cytokine levels and changes in ventilatory mechanics. Statistical analysis was conducted using the statistical software SPSS version 18.0 (SPSS, Chicago, IL, USA). A p-value (p) <0.05 was considered statistically significant.

## Results

No changes in physiological, hemodynamic, radiographic, or gasometric constants were observed (Table 1). These parameters were maintained within reference values throughout the duration of the study (p = NS ANOVA).

**Table 1:** Average arterial gasometric parameters.

	Group	Basal	Final	ANOVA
**pH**	**I**	**7.34**	**7.28**	**0.601**
	**II**	**7.37**	**7.33**	
**PCO_2_ (mmHg)**	**I**	**29.02**	**22.88**	**0.365**
	**II**	**24.86**	**26.20**	
**PO_2_ (mmHg)**	**I**	**138**	**193**	**0.738**
	**II**	**131**	**210**	
**FiO_2_**	**I**	**0.25**	**0.30**	**0.942**
	**II**	**0.28**	**0.30**	

### Plasma levels of TNF-α and IL-6

**Figure 1:**
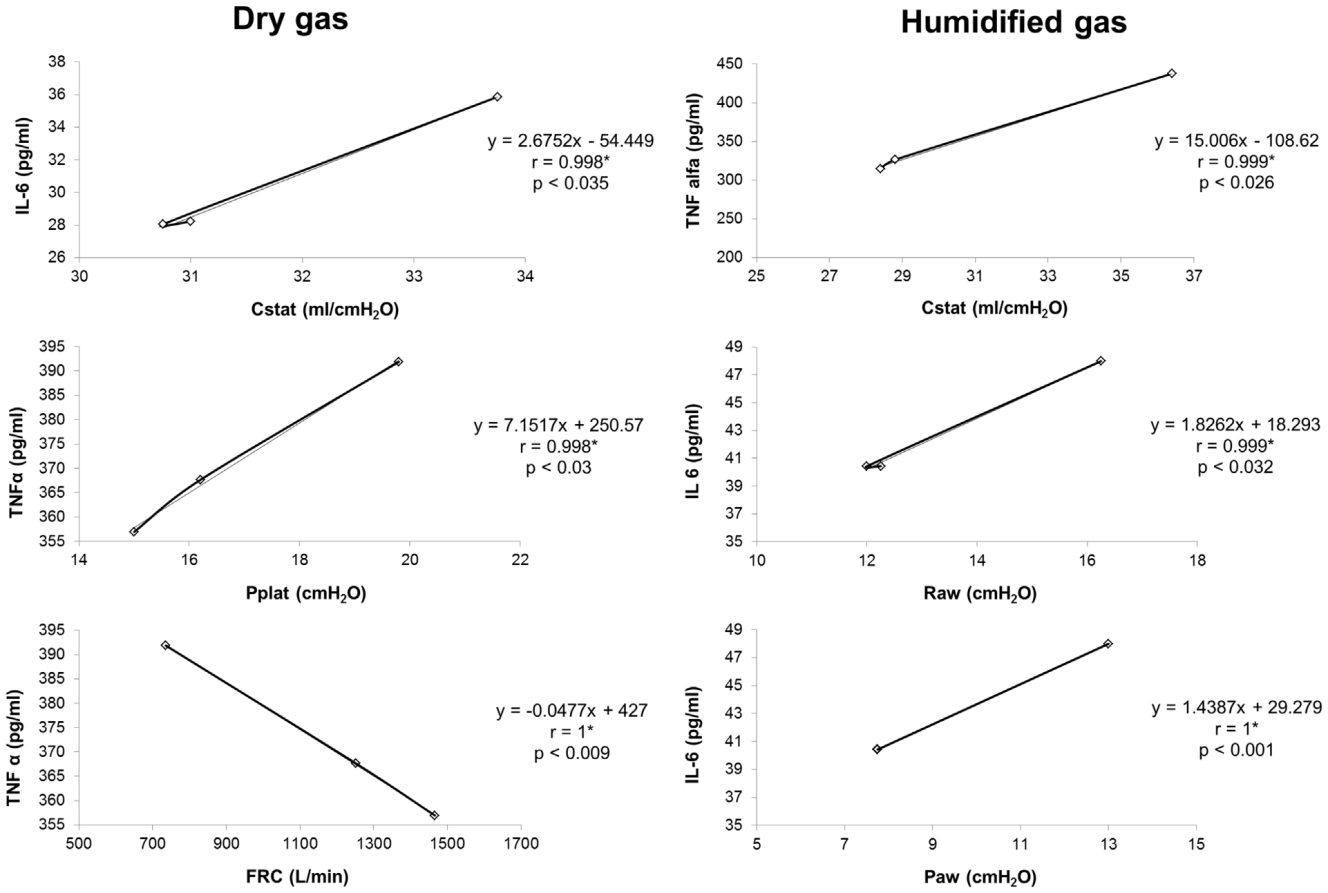
Correlation of interleukines with different ventilatory mechanics parameters. The correlation graphs and Pearson rank correlation coefficients (r values) are shown along with statistical significance of the correlation.

Plasma TNF-α levels increased in group I, reaching a maximum 24 hours after MV was initiated. There were no significant changes between groups (p = 0.77 ANOVA). However, this increase was significantly correlated to changes in TNF-α /Cstat (r = 0.999* p = 0.026); TNF-α /Pplat (r = 0.998* p = 0.03) and TNF-α /FRC (r = −1** p = 0.009) (Figure 1 and Table 2). Plasma IL-6 levels decreased at 12 hours and increased again towards the end of the study. There were no significant changes between groups (ANOVA p>0.05). IL-6 levels were only correlated to IL-6/Paw (r = 1* p = 0.001); IL-6/Raw (r = 0.999* p = 0.032) and IL-6/Cstat (r = 0.998* p = 0.035). There was no correlation between the levels of TNF-α and IL-6 in animals inhaling dry or humidified gases (Figure 1 and Table 2).

**Table 2:** Mechanical ventilation parameters and interleukin plasma levels.

	*Dry gases*		*Humidified gases*	
	*Basal*	*24 hours*	*Basal*	*24 hours*
***Cstat (ml/cmH_2_O)***	**36.4 (20–48)**	**28.4 (22–37)**	**33.7 (28–40)**	**31.0 (27–35)**
***Pplat (cmH_2_0)***	**15.0 (11–24)**	**19.8 (15–19.8)**	**18.0 (17–19)**	**22.0 (18–29)**
***Raw (cmH_2_O)***	**14.0 (11–17)**	**14.0 (6–25)**	**12.2 (3–16)**	**16.3 (15–17)**
***Paw (cmH_2_0)***	**8.0 (5–15)**	**9.2 (5–17)**	**7.7 (7–9)**	**9.0 (8–12)**
***PIP (cmH_2_0)***	**15.6 (1–24)**	**17.6 (12–21)**	**15.7 (14–18)**	**20.3 (19–22)**
***FRC (L/min)***	**1466.0 (910–2374)**	**735.0 (205–1185)**	**1193.5 (515–2694)**	**881.8 (359–1829)**
***TNF-α (pg/ml)***	**356.9 (24–1301)**	**391.9 (55–1283)**	**437.5 (36–1148.50)**	**314.9(36–992)**
***IL-6 (pg/ml)***	**35.9 (0–72)**	**28.2 (0–56)**	**40.4 (8–132)**	**47.9 (0–107)**

(Averages ± standard deviation.)

### Ventilatory mechanics findings

**Figure 2:**
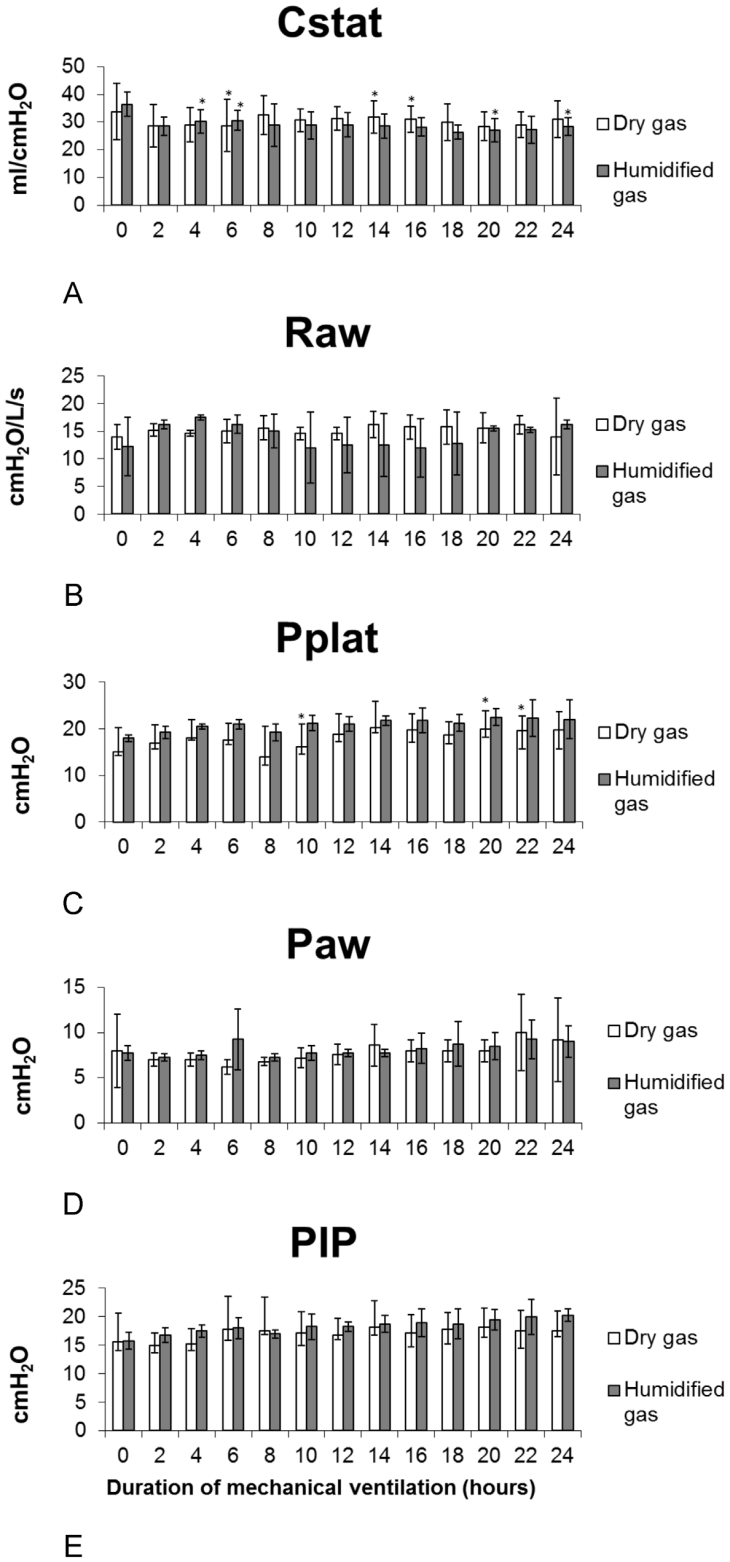
Averages ± standard deviations of the pulmonary mechanics during the time of study (24 hours) for both study groups. Compliance (Cstat), airway resistance (Raw), plateau pressure (Pplat), peak inspiratory pressure (PIP), mean airway pressure (Paw) (*p<0.05 ANOVA).

Cstat decreased in both groups starting two hours after MV was initiated, and the decrease was maintained until the end of the study (p = 0.90, ANOVA) (Figure 2.A and Table 2).

Raw values in both groups showed an increase compared to baseline values two hours after ventilation was initiated. In group I, this increase was maintained until the end of the study. In contrast, in group II, Raw values decreased at 10–18 hours, after which they increased again. However, these changes were not statistically significant either within or between the groups (p = 0.87 ANOVA) (Figure 2.B and Table 2).

Pplat increased in both groups two hours after ventilation was initiated (p = 0.02 ANOVA). In group I, Pplat increased starting at two hours post-MV, decreased at hour eight, and rose again until the end of the study, reaching its maximum at hour 20 (Figure 2.C and Table 2).

Paw behaved similarly in the two groups; it decreased two hours post-MV, returning to reference values throughout the rest of the study. No significant differences were found between groups (p = 0.90 ANOVA) (Figure 2.D and Table 2).

PIP increased in both groups beginning at two hours post-ventilation. However, this increase was not statistically significant (p = 0.67 ANOVA) (Figure 2.E and Table 2).

**Figure 3:**
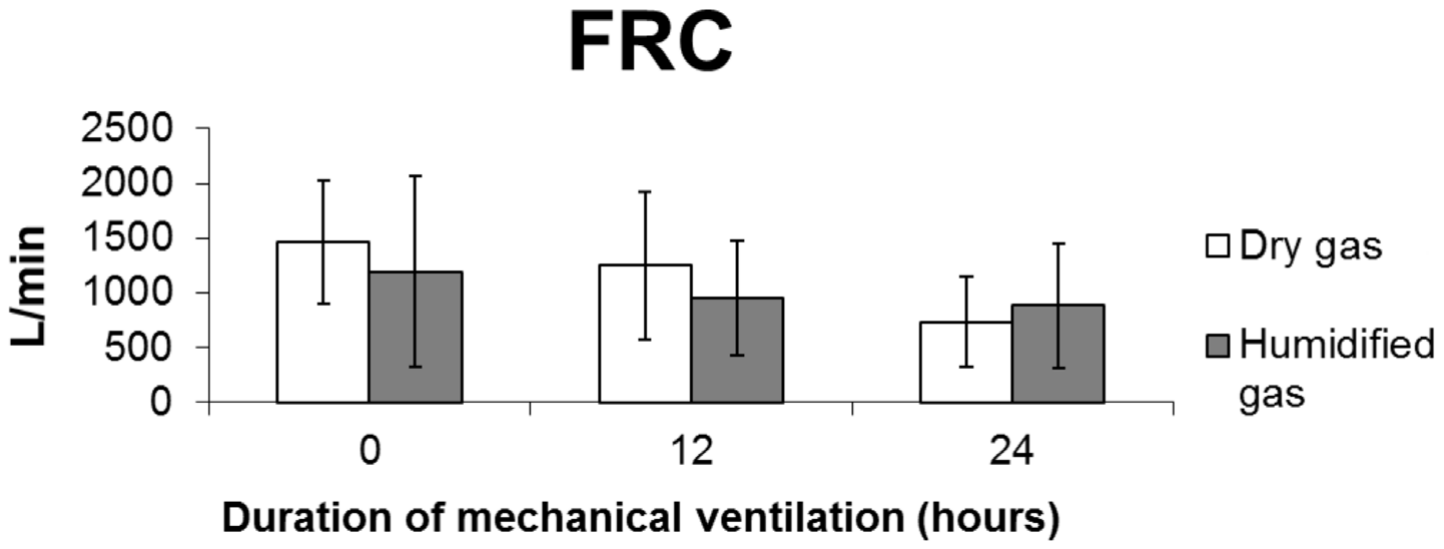
Averages ± standard deviations of the functional residual capacity (FRC) during the time of study (24 hours) for both study groups.

FRC decreased in both groups at 12 and 24 hours post-ventilation (p = 0.46 ANOVA), with a more pronounced decrease in group I (Figure 3 and Table 2).

### Microscopy

In the tracheal tissue, the following changes were observed in group I: four animals (80%) showed grade 1 neutrophil infiltration, two animals (40%) showed grade 1 lymphocyte infiltration, one animal (20%) showed grade 2 and one animal (20%) showed grade 1 hemorrhage of the tracheal wall, and one animal (20%) showed grade 1 epithelial necrosis.

**Figure 4:**
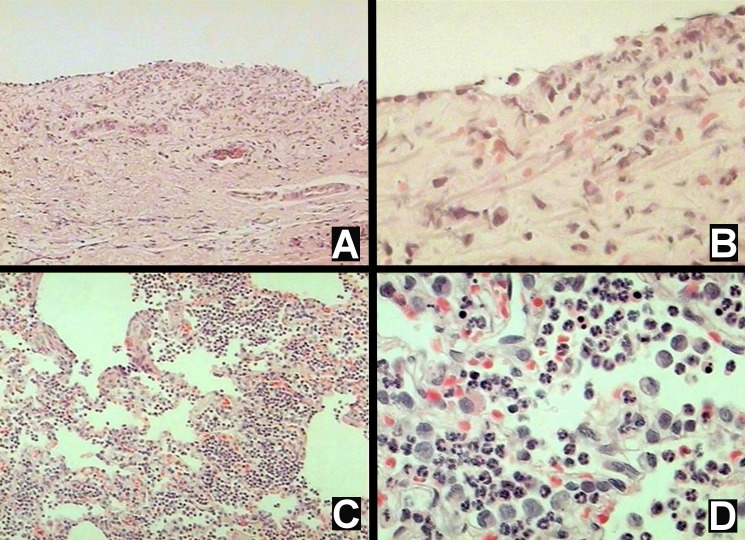
Micrograph of lung tissue stained with hematoxylin and eosin. A) and B) grade 2 inflammatory infiltration and grade 1 hemorrhage (10x and 40x magnification, respectively). C) and D) respiratory epithelium (main carina) that shows epithelial necrosis, with chromatolysis and hypereosinophilia of the cytoplasm (10x and 40x magnification, respectively).

In the main carina epithelium, the following changes were observed for group I: 20% of the animals showed grade 3 and 20% showed grade 1 neutrophil infiltration, and 20% of the animals showed epithelial necrosis (Figures 4A and 4B).

In the bronchial tissue, the following changes were observed for group I: one animal (20%) developed grade 2 and another animal (20%) developed grade 1 neutrophil infiltration.

In the pulmonary tissue, the following changes were observed for group I: 20% of the animals developed grade 1 alveolar edema, 80% developed grade 1 and 20% developed grade 2 hemorrhage, and 20% showed grade 1 neutrophil and macrophage infiltration (Figures 4C and 4D).

The only changes observed for group II were the following: 20% of the animals showed grade 1 neutrophil infiltration in the tracheal wall, and 20% showed grade 1 alveolar hemorrhage in the pulmonary tissue.

## Discussion

When gases are delivered through an endotracheal tube, there is a dehydrating effect on the inhaled air and an increase in airway mucus viscosity, increasing the risk of serious airway complications. Despite knowledge of this effect, few studies have evaluated the impact of MV on airway inflammation. In the present study, the effect of humidification of gases supplied by MV on plasma levels of TNF-α and IL-6 was assessed in dogs. The relationship between humidification and radiological, hemodynamic, gasometric, and microscopic changes of pulmonary mechanics was also investigated. This was achieved by measuring and comparing these parameters in dogs subjected to short-term MV with and without humidification.

TNF-α and IL-6 are chemoattractant molecules that cause neutrophil infiltration and exacerbate the inflammatory response. In the present study, TNF-α levels increased in animals subjected to MV without humidification. This can be compared to the findings of Pillow et al. [17], who found an increase in pro-inflammatory cytokine mRNA expression in lung tissue 3 hours after hyperoxic ventilation with cold and dry air without affecting ventilation mechanics. Tremblay et al. [9] reported an increase in TNF-α, IL-1β, IL-6, MIP-2, and IL-10 cytokine levels in the bronchoalveolar lavage obtained from isolated, non-perfused rat lungs ventilated for 2 hours. Von Bethmann et al. [18] also detected TNF-α and IL-6 in the perfusion liquid from isolated rat lungs that were ventilated with high volumes. These results suggest that TNF-α and IL-6 play important roles in the initiation and/or dissemination of systemic inflammatory responses after MV. Our data suggest that non-humidified MV can induce limited inflammation in previously healthy airways. Similarly, the increase in plasma levels of TNF-α and IL-6 shows a statistically significant correlation with changes in Pplat, FRC, and Cstat.

The decrease in Cstat in group I probably occurred because the gas was not able to pass through the upper airway. This caused loss of humidity in the upper respiratory tract and, consequently, its dehydration. When dry gas reaches the gas exchange zone, the production of surfactant is affected. This, in turn, decreases the alveolar surface tension and generates atelectasis zones, dry secretions, and airway obstruction. Together, these effects alter the pulmonary resistance and, consequently, the Cstat. Greenspan et al. [19] showed that five minutes of breathing dry and cold ambient gas caused a decrease in pulmonary Cstat in ventilated infants. The decrease in Cstat values observed in the group I animals is consistent with results reported by Rashad et al. [20], Fonkalsrud et al. [21], and Marfatia et al. [22], who studied the effect of MV with and without gas humidification in rabbits and dogs. They found that Cstat decreases when dry gas is administered. Our results are also in agreement with those of Sottiaux [23], who showed that changes in Cstat, Raw, and FRC could occur due to dry gas as well as to over-humidified gas.

There are several peculiarities in the way dogs disseminate heat compared to humans. Dogs essentially do not have sweat glands; instead, they sweat and disseminate heat primarily through the respiratory system through evaporation during panting. When a dog is subjected to general anesthesia and invasive MV, its panting capacity is lost. This limits the evaporation of humidity, causing an increase in the relative humidity of the upper airway. We can thereby speculate, although the humidification levels were not measured, that the decreased Cstat in group II animals in this study may have originated because of such an increase in airway humidity; the excess humidity in the pulmonary parenchyma occupies space, leading to surfactant dilution and a decrease in surface tension, factors that can induce alveolar collapse. Additionally, Froese and Bryan [24] showed that the effects of anesthetic gas administration and dorsoventral position can cause changes in pulmonary Cstat due to displacement of the diaphragm.

The increase in Raw values in group I animals was most likely caused by the lack of humidity, which may have resulted in dehydration of the respiratory tract (and a decrease in Cstat), decreased mucus production, increased mucus viscosity, and mucus clogging, all of which can cause changes in airflow and atelectasis. The increased Raw values observed in the present study are consistent with the results described by Cohen et al. [25], who indicated that application of dry and cold gases can cause lesions in the respiratory tract. The appearance of lesions affects the mobility of the tracheobronchial epithelium and its secretions, and causes airway obstruction. Tabka et al. [26] showed that healthy children exposed to cold temperatures and dry environments suffer exercise-induced bronchoconstriction and that Raw values are increased under these conditions. In a clinical study, Doyle et al. [27] compared the effects of a heat and humidity exchanger and showed that both generated inappropriate airway humidification, which in turn caused a high incidence of tracheal tube occlusion. Additionally, cold air induces a bronchoconstriction response, which is associated with muscarinic receptors in the nasal mucosa [28]. The increase in Raw values in group II could have been related to an increase in mucus viscosity due to over-humidification. This could have affected ciliary function, causing airway obstruction and, consequently, an increase in resistance [29].

The Pplat corresponds to the pressure at which alveolar pressure, which is required to maintain the elastic recoil of the respiratory system [30], can be indirectly assessed. Given that Pplat represents the equilibrium pressure after a pause in inhalation of at least 5 seconds, it is not modified by secretions, bronchospasm, or alterations in Raw values. However, Pplat does change in response to changes in pulmonary or thoracic Cstat and FRC. Our results are consistent with this; the decrease in Cstat in group I coincided with an increase in Pplat throughout the MV period. This increase was smaller for group II, in which the animals were ventilated with humidified gas.

Paw can be altered by increasing the flow volume and the PEEP. However, these parameters were not affected in the present study. Although variations were observed throughout the duration of the experiment, the values were maintained within the reference ranges [31].

PIP measurement has been standardized to avoid pneumothorax, pneumomediastinum, interstitial emphysema, pneumopericardium, and barotrauma. However, few studies mention its importance or describe methods for preventing its increase under normal conditions. Rozé et al. [32] showed that a decrease in PIP observed in the circuit is not necessarily associated with a decrease in airway diameter. Group I, which received dry gas, presented PIP variations. However, these were maintained within reference values, while the group II, which received humidified gas, showed a modest increase in PIP without modifications in lung mechanics.

The FRC is used as an indicator of the recruiting and de-recruiting states. To our knowledge, this is the first experimental study to use the Engstrom Carestation ventilator coupled to a humidity chamber to conduct FRC measurements in animals. This method for measuring FRC was validated by Olegård et al. [33], who showed that highly precise measurements could be obtained by using the nitrogen elimination method and changing the flow of oxygen/air, and by Dellamonica et al. [34], who found acceptable accuracy and precision for lung-volume measurement at different PEEP levels in patients with ARDS. Our results show that the use of dry gases during short-term MV causes a decrease in FRC values, in agreement with the findings of Sottiaux [23]. The latter author showed that FRC values change due to the use of dry and over-humidified gas. Over-humidification could explain the moderate decrease in FRC observed in the group II animals. The decrease in FRC, which was more evident in the group that received ventilation with dry gases, possibly suggests that pulmonary collapse occurs in both groups, but that it occurs earlier and more prominently in animals that do not receive humidified gas.

Damage to the tracheobronchial epithelium has been described in studies that compared the effects of dry gas and humidified gas using various experimental models. It has been observed that lack of humidification causes changes in the respiratory epithelium. After ventilating rabbits with dry gases, Marfatia et al. [22] found destruction of cilia and mucus glands, disruption and thinning of the basal membrane, and ulceration of the epithelium, all primarily within the upper airway. However, alterations in the pulmonary parenchyma were not studied. Using cultured epithelial cells exposed for four or eight hours to a dry environment, Chidekel et al. [35] found abnormal morphology, prominent nuclei, intracellular and nuclear vacuoles, diffuse cytoplasm, and cellular debris, while cells exposed to a humidity chamber did not show any obvious changes. The results of the current study suggest that inhalation of dry gas causes histological lesions in the entire respiratory tract, including pulmonary parenchyma, to a greater extent than humidified gas.

## Conclusions

In dogs under experimental conditions, after 24 hours of MV with dry gases, there is a non-significant increase in plasma levels of TNF-α and IL-6 for both groups. However, significant changes in Pplat were found in group I, and the decrease in FRC was more pronounced during MV with dry gases. Histological damage to respiratory epithelium by short-term MV was less marked in dogs that received humidified gases. The present study highlights the relevance of humidification in decreasing the severity of damage associated with MV. Our study shows that the cellular lesion induced by dry gases causes minimal inflammation, which in turn affects the respiratory mechanics.

## References

[1] Lellouche F, Maggiore SM, Deye N, Taillé S, Pigeot J, et al (2002) Effect of the humidification device on the work of breathing during noninvasive ventilation. Intensive Care Med 28: 1582–1589. 12415444 10.1007/s00134-002-1518-9

[2] Branson RD (2006) Humidification of respired gases during mechanical ventilation: mechanical considerations. Respir Care Clin N Am 12: 253–261. 16828693 10.1016/j.rcc.2006.03.011

[3] Goldsmith A, Shannon A (2009) Humidification devices. Anaesth Intens Care Med 10: 465–467.

[4] Parsons PE, Matthay MA, Ware LB, Eisner MD (2005) Elevated plasma levels of soluble TNF receptors are associated with morbidity and mortality in patients with ALI. Am J Physiol Lung Cell Mol Physiol 288: L426–L431. 15516488 10.1152/ajplung.00302.2004

[5] Arancibia F, Soto R (2010) Daño pulmonar inducido por la ventilación mecánica. Rev Chil Med Int 25: 205–210.

[6] Mercke U (1975) The influence of varying air humidity on mucociliary activity. Acta Otolaryngol 79: 133–139. 1146532 10.3109/00016487509124665

[7] Dorsch JA, Dorsch SE (1999) Anesthesia ventilators. In Dorsch JA, Dorsch SE, editors. Understanding Anesthesia Equipment. Baltimore: Williams & Wilkins, pp. 309–353.

[8] Gorayb SB, Braz JR, Martins RH, Módolo NS, Nakamura G (2004) Inhaled gases humidification and heating during artificial ventilation with low flow and minimal fresh gases flow. Rev Bras Anestesiol 54: 20–36. 19471707 10.1590/s0034-70942004000100004

[9] Tremblay LN, Miatto D, Hamid Q, Govindarajan A, Slutsky AS (2002) Injurious ventilation induces widespread pulmonary epithelial expression of tumor necrosis factor α and interleukin-6 messenger RNA. Crit Care Med 30: 1693–1700. 12163778 10.1097/00003246-200208000-00003

[10] Haitsma JJ, Uhlig S, Verbrugge SJ, Göggel R, Poelma DL, et al (2003) Injurious ventilation strategies cause systemic release of IL-6 and MIP-2 in rats in vivo. Clin Physiol Funct Imaging 23: 349–353. 14617266 10.1046/j.1475-0961.2003.00518.x

[11] SAGARPA (Secretaría de Agricultura, Ganadería, Desarrollo Rural, Pesca y Alimentación) (1999) Especificaciones Técnicas para la Producción, Cuidado y Uso de Animales de Laboratorio de la Norma Oficial Mexicana NOM-062-ZOO-1999. Mexico City. Diario Oficial de la Federación. 58 pp.

[12] NIH (National Institutes of Health U.S.A) (2011) Guide for the Care and Use of Laboratory Animals. Washington, D.C. The National Academies Press.246 pp.

[13] Balls M (1994) Replacement of animal procedures: alternatives in research, education and testing. Lab Anim 28: 193–211. 7967458 10.1258/002367794780681714

[14] Kilkenny C, Browne WJ, Cuthill IC, Emerson M, Altman DG (2010) Improving Bioscience Research Reporting: The ARRIVE Guidelines for Reporting Animal Research. PLoS Biol 8: e1000412 doi:10.1371/journal.pbio.1000412 20613859 10.1371/journal.pbio.1000412 PMC2893951

[15] Lellouche F, Taillé S, Maggiore SM, Qader S, L'her E, et al (2004) Influence of ambient and ventilator output temperatures on performance of heated-wire humidifiers. Am J Respir Crit Care Med 170: 1073–1079. 15271695 10.1164/rccm.200309-1245OC

[16] Koksel O, Kaplan MB, Ozdulger A, Tamer L, Degirmenci U, et al (2005) Oleic acid-induced lung injury in rats and effects of caffeic acid phenethyl ester. Exp Lung Res 31: 483–496. 16019983 10.1080/01902140590918876

[17] Pillow JJ, Hillman NH, Polglase GR, Moss TJ, Kallapur SG, et al (2009) Oxygen, temperature and humidity of inspired gases and their influences on airway and lung tissue in near-term lambs. Intensive Care Med 35: 2157–2163. 19756508 10.1007/s00134-009-1624-z

[18] Von Bethmann AN, Brasch F, Nüsing R, Vogt K, Volk HD, et al (1998) Hyperventilation induces release of cytokines from perfused mouse lung. Am J Respir Crit Care Med 157: 263–272. 9445308 10.1164/ajrccm.157.1.9608052

[19] Greenspan JS, Wolfson MR, Shaffer TH (1991) Airway responsiveness to low inspired gas temperature in preterm neonates. J Pediatr 118: 443–455. 1999789 10.1016/s0022-3476(05)82165-1

[20] Rashad K, Wilson K, Hurt HH, Graff TD, Benson DW (1967) Effect of humidification of anesthetic gases on static compliance. Anesth Analg 46: 127–133. 6066847

[21] Fonkalsrud EW, Sanchez M, Higashijima I, Arima E (1975) A comparative study of the effects of dry vs. humidified ventilation on canine lungs. Surgery 78: 373–380. 1173950

[22] Marfatia S, Donahoe PK, Hendren WH (1975) Effect of dry and humidified gases on the respiratory epithelium in rabbits. J Pediatr Surg 10: 583–592. 1185448 10.1016/0022-3468(75)90360-7

[23] Sottiaux TM (2006) Consequences of under -and over-humidification. Respir Care Clin N Am 12: 233–252. 16828692 10.1016/j.rcc.2006.03.010

[24] Froese AB, Bryan AC (1974) Effects of anesthesia and paralysis on diaphragmatic mechanics in man. Anesthesiology 41: 242–255. 4604401 10.1097/00000542-197409000-00006

[25] Cohen LL, Weinberg PF, Fein IA, Rowinski GS (1988) Endotracheal tube occlusion associated with the use of heat and moisture exchangers in the Intensive care unit. Crit Care Med 16: 277–279. 3422625 10.1097/00003246-198803000-00013

[26] Tabka Z, Ben Jebria A, Vergeret J, Guenard H (1988) Effect of dry warm air on respiratory water loss in children with exercise-induced asthma. Chest 94: 81–86. 3383660 10.1378/chest.94.1.81

[27] Doyle A, Joshi M, Frank P, Craven T, Moondi P (2011) A change in humidification system can eliminate endotracheal tube occlusion. J Crit Care 26: 637.e1–4. 10.1016/j.jcrc.2011.02.004 21439765

[28] On LS, Boonyongsunchai P, Webb S, Davies L, Calverley PM, et al (2001) Function of pulmonary neuronal M(2) muscarinic receptors in stable chronic obstructive pulmonary disease. Am J Respir Crit Care Med 163: 1320–1325. 11371395 10.1164/ajrccm.163.6.2002129

[29] Modell JH, Giammona ST, Davis JH (1967) Effect of chronic exposure to ultrasonic aerosols in the lung. Anesthesiology 28: 680–688. 6028058 10.1097/00000542-196707000-00013

[30] Pérez M, Mancebo J (2006) Monitoring ventilatory mechanics. Med Intensiva 30: 440–448. 17194401 10.1016/s0210-5691(06)74567-3

[31] Stewart TE, Meade MO, Cook DJ, Granton JT, Hodder RV, et al (1998) Pressure- and Volume-Limited Ventilation Strategy Group: Evaluation of a ventilation strategy to prevent barotrauma in patients at high risk for acute respiratory distress syndrome. N Eng J Med 338: 355–361. 10.1056/NEJM199802053380603 9449728

[32] Rozé H, Lafargue M, Batoz H, Picat MQ, Perez P, et al (2010) Pressure-controlled ventilation and intrabronchial pressure during one-lung ventilation. Br J Anaesth 105: 377–381. 20554634 10.1093/bja/aeq130

[33] Olegård C, Söndergaard S, Houltz E, Lundin S, Stenqvist O (2005) Estimation of functional residual capacity at the bedside using standard monitoring equipment: a modified nitrogen washout/washin technique requiring a small change of the inspired oxygen fraction. Anesth Analg 101: 206–212. 15976233 10.1213/01.ANE.0000165823.90368.55

[34] Dellamonica J, Lerolle N, Sargentini C, Beduneau G, Di Marco F, et al (2011) Accuracy and precision of end-expiratory lung-volume measurements by automated nitrogen washout/washin technique in patients with acute respiratory distress syndrome. Crit Care 15: R294. 22166727 10.1186/cc10587 PMC3388680

[35] Chidekel A, Zhu Y, Wang J, Mosko JJ, Rodriguez E, et al (2012) The effects of gas humidification with high-flow nasal cannula on cultured human airway epithelial cells. Pulm Med 2012: 380686. 22988501 10.1155/2012/380686 PMC3439979

